# The MHC-I related protein 1 MR1 can bind host purine catabolites

**DOI:** 10.1016/j.jbc.2026.113428

**Published:** 2026-08-12

**Authors:** Mohamed R. Abdelaal, Xin Yi Lim, Jeffrey Y.W. Mak, David P. Fairlie, James McCluskey, Alexandra J. Corbett, Nicholas A. Gherardin, Wael Awad, Jamie Rossjohn

**Affiliations:** 1 Department of Biochemistry and Molecular Biology, Immunity Program, Biomedicine Discovery Institute, Monash University, Clayton, Australia; 2 Department of Microbiology and Immunology, The University of Melbourne at the Peter Doherty Institute for Infection and Immunity, Melbourne, Australia; 3 Centre for Chemistry and Drug Discovery and ARC Centre of Excellence for Innovations in Peptide and Protein Science, Institute for Molecular Bioscience, The University of Queensland, Brisbane, Queensland, Australia

**Keywords:** MR1, mucosal-associated invariant T cell, antigen presentation, nucleobases, structural immunology

## Abstract

The major histocompatibility complex class I–related molecule (MR1) is a non-classical antigen-presenting protein that presents a diverse array of microbial, synthetic and host-generated small metabolites to mucosal-associated invariant T (MAIT) cells and other MR1-restricted T (MR1T) cell populations. Recent studies showed that MR1 binds carbonyl-nucleobase adducts, resulting in the activation of some MR1T cell clones. However, whether endogenous canonical nucleobases and their derivatives can impact the MR1 axis remains unclear. Here, through biochemical and cellular screening of a library of canonical nucleobases and their catabolites, we demonstrate that the catabolites of purine degradation, namely uric acid, xanthine, and xanthosine, can, at high concentrations, bind MR1 and promote its intracellular retention, resulting in reduced MR1 surface expression. These purine-based nucleobases can compete with microbial and non-microbial ligands for MR1 binding. Analysis of three crystal structures of ligand-bound MR1 shows that these endogenous purine-based nucleobases adopt distinct modes of binding and interactions within the MR1 A′-pocket. Accordingly, we broaden the repertoire of MR1-ligandome by describing a group of host xanthine-based compounds that can bind MR1 and regulate its trafficking to the cell surface.

One of the key elements of adaptive immune responses is the capacity of T lymphocytes to recognise a variety of antigens (Ag) through the cognate co-engagement of their T cell receptors (TCRs) with Ags presented by Major Histocompatibility Complex (MHC) molecules and MHC-like molecules, on the surface of antigen-presenting cells (1). Chemically diverse ligands include peptide fragments produced by the proteolytic degradation of self or foreign proteins within cells expressing MHC-I and MHC-II molecules (2). In addition, the MHC I-like CD1 antigen-presenting molecules present self and foreign cellular lipids to CD1-restricted T cells (3). MHC class I-related molecule (MR1) displays small-molecule metabolites that are derived from either microbial or non-microbial origins to diverse MR1-restricted T cells (MR1T), including mucosal-associated invariant T (MAIT) cells, which are abundant in humans (4, 5, 6, 7). MAIT cells are an innate-like T cell population that are involved in antimicrobial immunity, autoimmunity (8), wound repair (9) and anti-tumor immunity (10).

The evolutionarily conserved MR1 molecule is ubiquitously expressed by all nucleated cells but its cell surface expression is strictly dependent on Ag availability (11, 12). Six different MR1 allomorphs have been characterized where MR1∗01 is the predominant form expressed in humans (13, 14). Although MR1 has two potential Ag-binding pockets (A′- and F′-pockets), all MR1 ligands identified to date are sequestered within the MR1 A′-pocket. Of note, several MR1 ligands are stabilized within the pocket by forming a Schiff base bond with the MR1-Lys43 residue, and this is important in allowing MR1 to egress to the cell surface (4, 11). However, MR1 cell surface upregulation is not strictly dependent on the Schiff base formation, as seen with several ribityl-lumazine ligands that did not create a Schiff base with MR1 but were able to induce modest MR1 upregulation (15).

The MR1 ligandome includes ligands that originate from outside the host (*i*.*e*., exogenous), including microbial and non-microbial ligands as well as host-derived metabolites (16). MR1 presents microbial derivatives of vitamin B2 (riboflavin) precursors, which are produced by a wide range of riboflavin-producing microbes (17, 18). The microbial intermediate 5-amino-6-D-ribitylaminouracil (5-A-RU) non-enzymatically reacts with small glycolysis metabolites (*e*.*g*., methylglyoxal) to generate short-lived intermediate antigens such as 5-(2-oxopropylideneamino)-6-D-ribitylaminouracil (5-OP-RU), which are captured by MR1 or otherwise undergo cyclization forming the more stable ribityllumazines, including 7-methyl-ribityllumazine (RL-7-Me) (17). These antigens contain a ribityl moiety, which is required for MAIT cell activation, but have a wide range of activation potency (17, 18, 19). MR1 molecules can also present non-microbial ligands including photo-degradation products of folic acid (vitamin B9); 6-formylpterin (6-FP), and its synthetic derivative, acetyl-6-FP (Ac-6-FP) (4, 20), as well as a diverse panel of diet, drug and drug-like molecules, synthetic aromatic metabolites and environmental ligands (11, 12, 21, 22, 23, 24, 25). Most of these ligands increase cell surface MR1 expression when added to MR1-expressing cells *in vitro*. Recent studies reported MR1 ligands that originate from the host itself, including vitamin B6 vitamers and bile acid derivatives (26, 27). Furthermore, we demonstrated that host-derived riboflavin catabolites, such as lumichrome, bind MR1, promote its retention within the ER, and limit its trafficking to the cell surface (28). These findings revealed an endogenous mechanism for regulating MR1 surface expression and, consequently, MR1-dependent immune responses. Furthermore, MR1 was recently reported to sense host nucleobase-based antigens, which are formed by either: (*i*) the condensation of endogenous nucleobases by stress-induced carbonyl-containing metabolites, such as 8-(9H-purin-6-yl)-2-oxa-8- azabicyclo[3.3.1]nona-3,6-diene-4,6-dicarbaldehyde [M_3_Ade], which is formed by the condensation of adenine by malondialdehyde metabolite (29, 30), or (*ii*) the direct oxidation of nucleobases resulting in the formation of oxidation products, such as 5-formyl-2′-deoxyuridine (5-FdU) and 5-formyluracil (5-FU) (31). Notably, 5-FdU and M_3_Ade have the capacity to stimulate various MR1T cells in an MR1-dependent manner; however, it is not clear whether typical nucleobases and their degradation products influence MR1-MR1T biology. Indeed, the full scope of the MR1 ligands diversity remain unclear.

In the present study, we systematically screened a panel of canonical nucleobases and related metabolites (a total of 14 compounds) for their ability to interact with MR1. We identified three xanthine-derived compounds as MR1-binding ligands that impair MR1 trafficking to the cell surface. Collectively, these findings expand the known repertoire of MR1 ligands and further support the concept that structurally diverse small molecules can regulate MR1 availability and function through the modulation of its intracellular trafficking.

## Results

### MR1 can capture uracil- and xanthine-based derivatives

**Figure 1:**
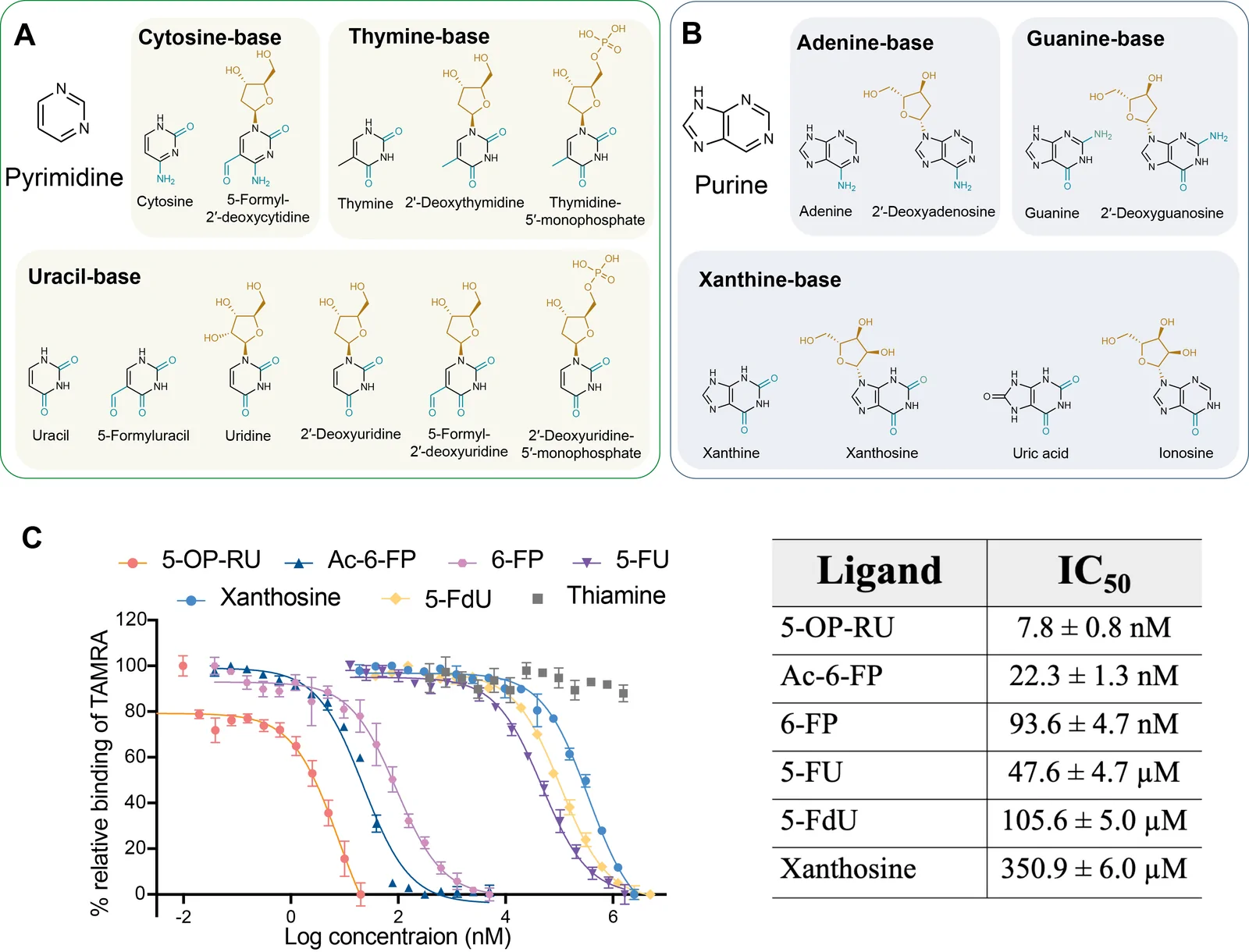
**Assessment of nucleobase-related compounds loading into MR1.***A* and *B*, chemical structures of pyrimidine (*A*) and purine-based compounds (*B*). *C*, titration curves of the ligand binding to MR1 were plotted from the non-linear regression analysis of the Fluorescence Polarisation assay data (*left*). These sigmoidal dose-response curves were plotted using GraphPad Prism software, enabling the calculation of IC_50_ values for each compound (*right*). Each data point represents normalised percentage binding from two independent experiments performed in triplicate. Mean values are plotted with SD represented in error bars.

In mammals, there are two classes of archetypical nucleobases: (*i*) pyrimidines-including cytosine, thymine, and uracil-which have a single nitrogenous ring as their primary structures (Fig. 1*A*); and (*ii*) purines-including adenine and guanine-which bear bicyclic rings (Fig. 1*B*). These nucleobases can be generated from DNA degradation and RNA turnover in the mammalian cell’s cytoplasm (32, 33). Other purine scaffolds can be formed in the cells during purine catabolism. These include xanthosine, xanthine, and uric acid compounds (Fig. 1*B*). Modern sequencing technologies have additionally revealed an increased abundance of post-transcriptionally modified nucleobases (34). These nucleobases have previously been identified as major byproducts of oxidative DNA and RNA damage, arising from enzymatic oxidation during cellular transformation and other nucleic acid modifications (35, 36, 37). For example, 5-FdU and 5-Formylcytidine (5-FdC) are oxidatively modified products of thymine and cytosine, respectively, which are preferentially repaired through lesion excision pathways rather than by nucleotide excision repair (38, 39).

Here, we assessed the capability of a panel of endogenous, naturally occurring, typical and modified nucleotide-related compounds (a total of 14 compounds) (Fig. 1, *A* and *B*) to bind MR1. First, we used a fluorescence polarization (FP)-based cell-free assay (25), in which a fluorescently tagged MR1 ligand (MAgA-TAMRA) was loaded into MR1 protein *in vitro* and competitively displaced by candidate ligands over a range of concentrations. This yields a direct evaluation of the ligand’s MR1 binding affinity, indicated by the measured IC_50_ value, without the complication of any cellular factors. Exploring the affinities of uric acid and xanthine toward MR1 was not feasible due to insolubility of the compounds in the experimental FP assay buffer. We found that the well-characterized ligands Ac-6-FP, 6-FP and 5-OP-RU, each of which can form a Schiff base with MR1, demonstrated the highest affinity (∼nM) toward MR1, consistent with previous measurements (28) (Fig. 1*C*). Additionally, thiamine, which did not show MR1 binding, was used as a negative control compound. The FP assay demonstrated that three nucleotide-related compounds (5-FU, 5-FdU and xanthosine) bind MR1 with different affinities (Fig. 1*C*). We recently showed that 5-FU and 5-FdU bind to the A′-pocket of MR1 (31). Despite their capacity to form a Schiff base with MR1, 5-FU and 5-FdU both displayed moderate affinities for MR1, with IC_50_ values of 47.6 ± 4.7 μM and 105.6 ± 5 μM, respectively, categorizing them as moderate MR1 binders. Additionally, xanthosine appeared to be a weak MR1 binder with an IC_50_ of 350 ± 6.0 μM, yet it has a higher affinity to MR1 than the non–Schiff based ligands such as the diclofenac derivatives (IC_50_ ∼ 2 mM) (25).

We next sought to investigate whether any of these candidate MR1 ligands were able to cause MR1 egress to the cell surface. To this end, Class I reduced (C1R^WT^) cells, a B cell lymphoblastoid cell line, were incubated for 3 h with various concentrations of the compounds in co-culture with the Jurkat.MAIT A-F7 TCR (TRAV1-2/TRBV6-1) reporter cells (17). Then, the cell surface level of MR1 on C1R cells and the surface expression of CD69, an activation marker, on Jurkat cells were simultaneously quantified by flow cytometry as performed previously (15, 20). Although some compounds possess a formyl group that would be predicted to form a Schiff base with Lys43, none of the tested compounds was found to upregulate MR1 on the cell surface when tested at concentrations up to 200 μM (Fig. S1). Instead, we observed a slight decrease in MR1 surface expression with three purine-based compounds that share the xanthine bicyclic scaffold (xanthosine, xanthine and uric acid) (Fig. S1*A*). The cell viability was not affected after treatment with the ligands. The slight MR1 downregulation appeared to be specific to MR1, as the surface expression of MHC-I was not altered by the addition of these compounds (Fig. S1*B*). In addition, none of the tested compounds induced the activation of Jurkat MAIT AF7 reporter cells within co-cultures (Fig. S1*C*). Thus, in subsequent experiments, we focused on characterizing the impact of xanthine-based compounds on MR1 biology.

### Xanthine-based compounds selectively reduce cell surface MR1

**Figure 2:**
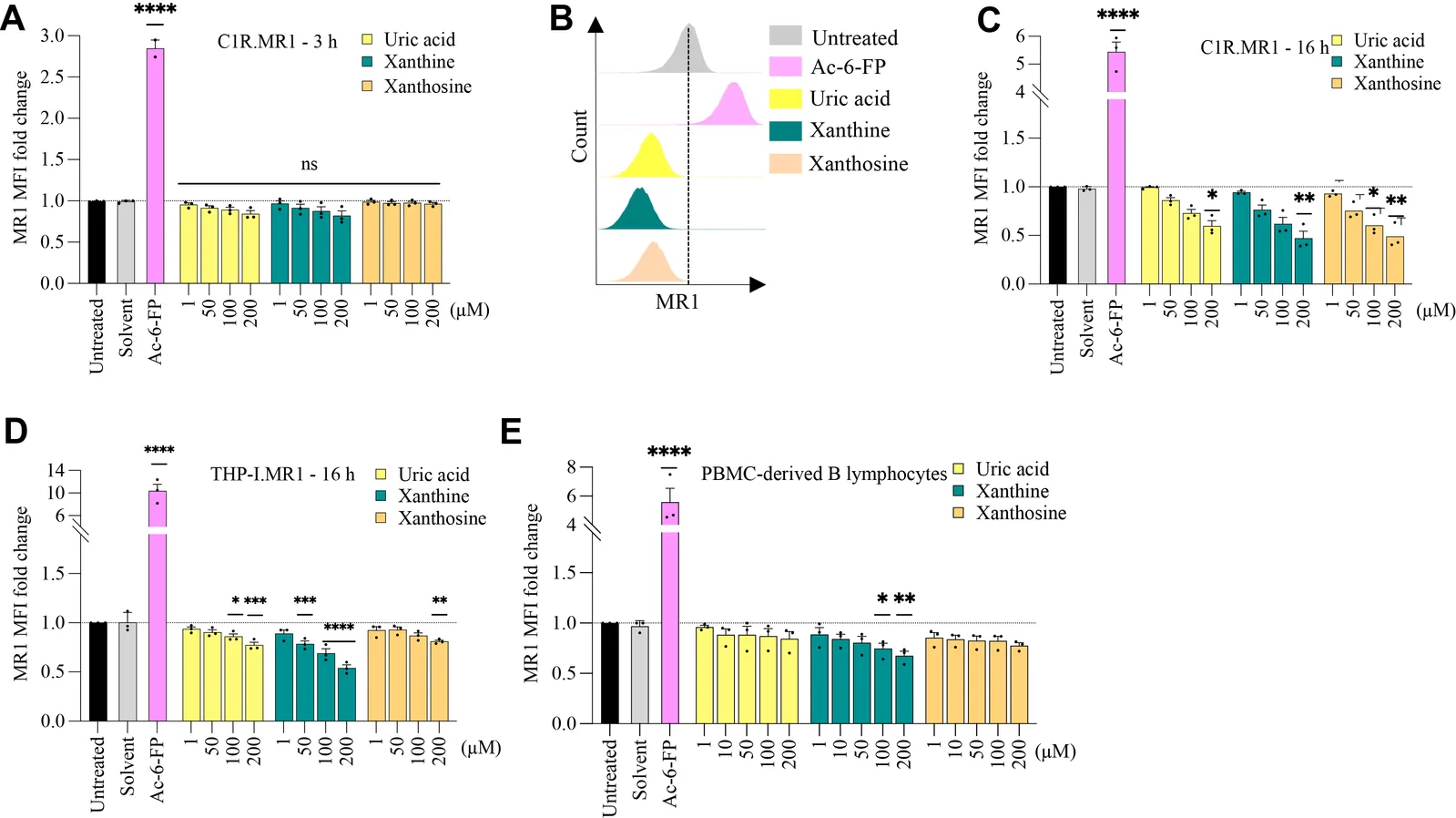
**Xanthine-based compounds impact on surface MR1 expression in different APCs.***A–C*, Bar graphs depict the expression of surface wild-type MR1 on C1R.MR1 cells incubated for 3 h (*A*) or 16 h (*B* and *C*) with titrated quantities of ligands, followed by staining with 8F2.F9 anti-MR1 antibody. *Panel B* shows overlay histograms for the expression of surface wild-type MR1 on C1R.MR1 cells after 16-h treatment with the highest dose of xanthine-based compounds. *D*, MR1 was quantified after adding the indicated concentrations of xanthine-based compounds to THP-1.MR1 cells. *E*, MR1 expression on the surface of PBMC-derived B lymphocytes was measured after treatment with xanthine-based compounds for 16 h. Data in (*A–D*) represent the fold change of the geometric mean fluorescence intensity (MFI) from three independent experiments performed in duplicates, with standard error (SEM) represented by the error bars. Data in (*E*) represent the average of three independent experiments performed on PBMCs from three independent donors in duplicates, with standard error (SEM) represented by the error bars. One-way ANOVA statistical analysis was performed for all samples, with Dunnett’s multiple comparisons performed using solvent as a control (ns: not significant, ∗*p* < 0.05, ∗∗*p* < 0.01, ∗∗∗*p* < 0.001, ∗∗∗∗*p* < 0.0001). MR1, MHC-I–related protein.

As C1R^WT^ cells have low basal surface MR1 levels, whose further reduction becomes undetectable by the anti-MR1 antibody, we sought to test the impact of a broad range of concentrations of xanthine-based compounds on MR1 expression on the surface of different MR1-overexpressing antigen-presenting cells. Here, C1R.MR1 cells were incubated with 1 to 200 μM of uric acid, xanthine and xanthosine, and the cell surface levels of MR1 were quantified by flow cytometry as shown above. Cell viability was unaffected, even at the highest concentrations of the catabolites (Fig. S2*A*). In C1R.MR1 cells, none of the xanthine-based compounds could upregulate MR1 surface expression after 3 or 16 h, which aligns with the co-culture results (Fig. 2, *A*–*C*). Although none of the tested compounds could significantly reduce the MR1 cell surface after 3 h (Fig. 2*A*), all xanthine-based compounds caused a decrease in the surface MR1 expression after 16 h at high concentrations (Fig. 2*C*). The cell surface downregulation of MR1 observed in C1R.MR1 cells was specific to MR1, as the level of HLA-A∗02:01 was not affected after incubating HLA-transduced C1R cells (C1R-A2) with the ligands (Fig. S2*B*). A similar trend was observed after incubating the MR1-overexpressing monocytic cell line THP-1.MR1 with xanthine-based compounds for 16 h (Fig. 2*D*). We next employed an assay system that uses primary APCs, which more closely mimic physiological antigen presentation conditions. Here, we treated PBMCs with xanthine-based compounds and quantified the level of cell surface MR1 using flow cytometry. The gating strategy for this experiment is shown in Fig. S2*C*. Here, the ligands modestly reduced MR1 expression on the surface of B lymphocytes, with only xanthine showing statistically significant downregulation (Fig. 2*E*). Overall, our data suggest that the direct interactions of xanthine-based compounds with MR1 in the cells lead to the reduction of cell surface expression.

### Requirements for xanthine compounds-mediated downregulation of cell surface MR1

**Figure 3:**
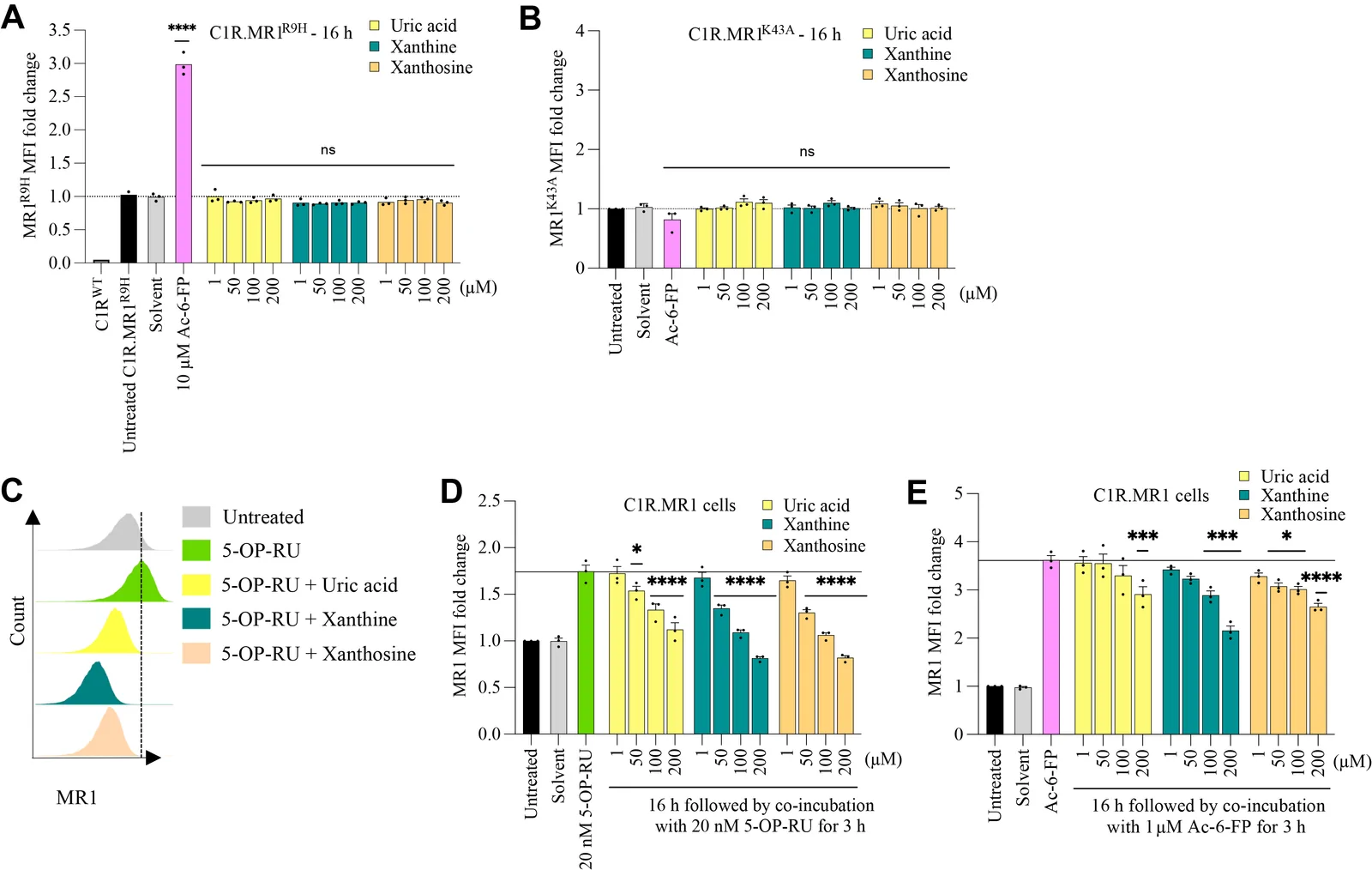
**Xanthine-based compounds induce intracellular retention of wild-type MR1, not MR1 mutants.***A* and *B*, C1R cells overexpressing MR1^R9H^ (*A*) or MR1^K43A^ mutants (*B*) were incubated for 16 h with titrated quantities of xanthine-based compounds, followed by flow cytometry. *C–E*, the competition between xanthine-based compounds and 5-OP-RU (*C* and *D*) or Ac-6-FP (*E*) in C1R.MR1 cells was quantified after incubation with the indicated concentrations of xanthine-based compounds for 16 h before the addition of Ac-6-FP or 5-OP-RU, as indicated, for a further 3 h. *Panel C* shows overlay histograms for the expression of surface wild-type MR1 on C1R.MR1 cells after treatment with the highest dose of xanthine-based compounds with 5-OP-RU. Data in *A* and *B* and *D* and *E* represent the fold change of MFI from three independent experiments performed in duplicate, with standard error (SEM) represented by error bars. One-way ANOVA statistical analysis was performed for all samples, with Dunnett’s multiple comparisons performed using solvent, Ac-6-FP, or 5-OP-RU as controls for the comparison (ns: not significant, ∗*p* < 0.05, ∗∗∗*p* < 0.001, ∗∗∗∗*p* < 0.0001). MFI, geometric mean fluorescence intensity.

We next investigated the impact of xanthine-based compounds on the surface expression of two MR1 mutants, MR1^R9H^ (40) and MR1^K43A^ (41). Both mutations stabilize MR1 and promote its trafficking to the cell surface, independent of ligand binding. The MR1^R9H^ variant, a rare naturally occurring human polymorphism, is unable to bind and present riboflavin-derived ligands such as 5-OP-RU (13), while retaining the capacity to bind alternative ligands, including Ac-6-FP and pyridoxal. This makes it a useful model to probe the molecular requirements for ligand binding. Using a C1R.MR1^R9H^ cell line, generated by stable transduction of C1R cells with the MR1^R9H^ construct (40), we assessed MR1^R9H^ surface expression upon co-culture with the xanthine-based compounds. Consistent with previous findings, C1R.MR1^R9H^ cells exhibited elevated baseline MR1 surface levels (Fig. 3*A*). As expected, Ac-6-FP treatment significantly increased MR1^R9H^ surface expression (Fig. 3*A*). In contrast, incubation with xanthine-based compounds did not alter MR1^R9H^ surface levels, indicating a lack of productive binding (Fig. 3*A*). Notably, treatment with xanthine-based compounds did not impact MR1^K43A^ surface expression (Fig. 3*B*). These findings are consistent with a model in which xanthine-based compounds bind MR1 but promote its intracellular retention in the wild-type context, thereby reducing surface expression.

### Xanthine-based compounds compete with MR1 ligands for binding to MR1

Next, we tested whether xanthine-based compounds modulate the presentation of potent microbial and non-microbial MR1 antigens. C1R.MR1 cells were preincubated with uric acid, xanthine or xanthosine overnight before the addition of Ac-6-FP or 5-OP-RU and coincubation for a further 3 h. The three compounds significantly inhibited the Ac-6-FP- and 5-OP-RU-induced MR1 upregulation in a concentration-dependent manner (Fig. 3, *C*–*E*). The competition with 5-OP-RU was more potent with the tested concentrations of the xanthine-based compounds (Fig. 3, *D* and *E*). These cellular data suggest that uric acid, xanthine and xanthosine can bind MR1 molecules within cells and hinder the capacity of other potent ligands to stimulate MR1 trafficking to the cell surface.

### Xanthine-based compounds do not competitively inhibit the activity of MAIT cells

**Figure 4:**
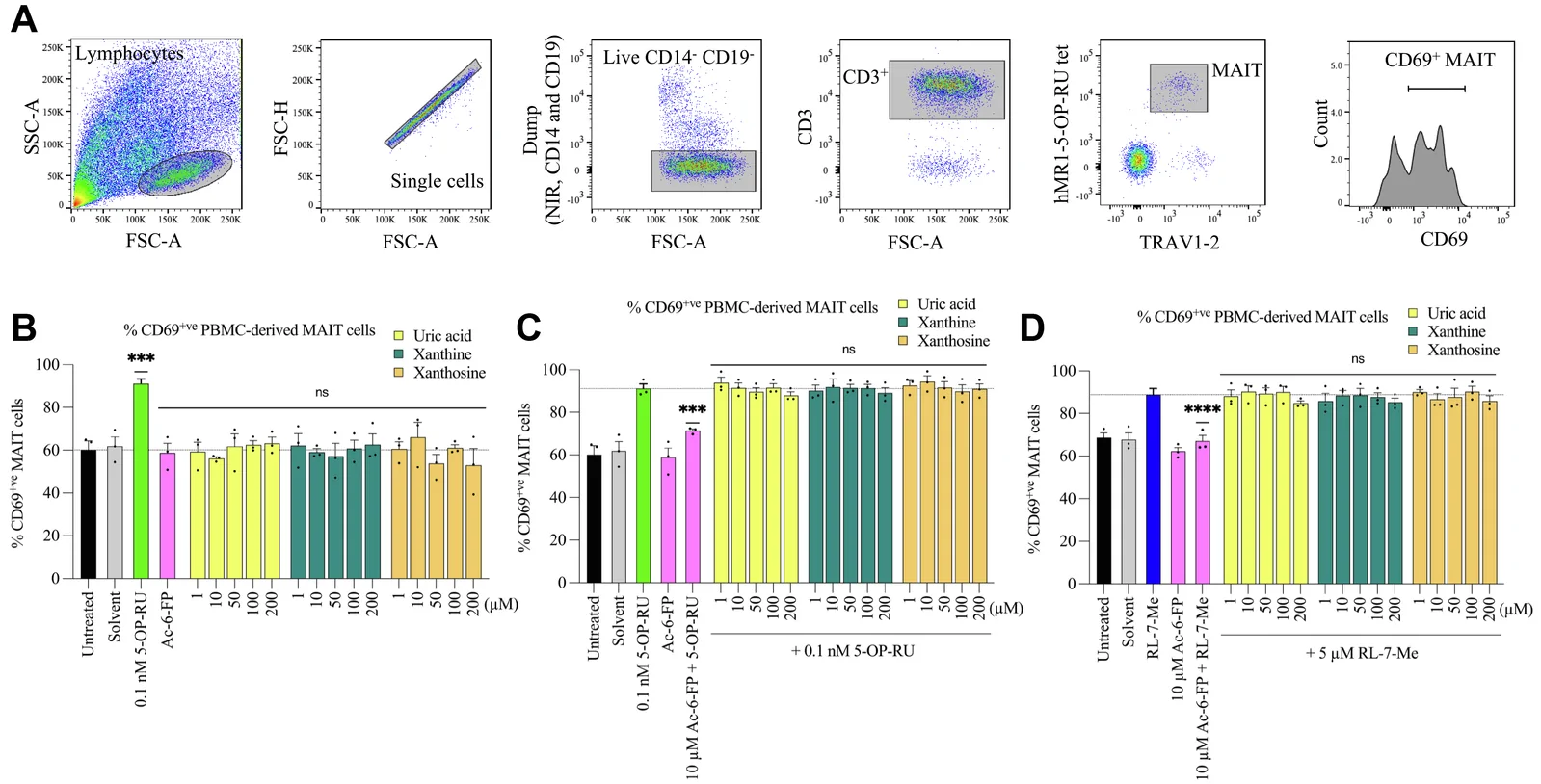
**Xanthine-based compounds do not competitively inhibit MAIT cell activation.***A*, gating strategy of PBMC inhibition experiments. *B–D*, Bar graphs show the proportion of CD69^+ve^ MAIT cells from PBMCs co-incubated with titrated doses of xanthine-based compounds in the absence (*B*) or presence of 5-OP-RU (*C*) or RL-7-Me (*D*). Data from all panels represent the mean of three independent experiments performed in duplicate on PBMCs from three different donors. The error bars represent the standard error of the mean (SEM). One-way ANOVA statistical analysis was performed for all samples, with Dunnett’s multiple comparisons performed using solvent, 5-OP-RU, or RL-7-Me as controls (ns: not significant, ∗∗∗*p* < 0.001, ∗∗∗∗*p* < 0.0001).

Given the intracellular MR1 retention resulting from the addition of xanthine-based compounds, we anticipated that these ligands could influence the activation of MAIT cells. Here, we employed an assay system incorporating primary antigen-presenting cells and MAIT cells to more closely recapitulate physiological antigen presentation (28). In this approach, healthy human PBMCs were first co-cultured with xanthine-based compounds alone, and MAIT cell responses were assessed. MAIT cell activation was quantified by measuring CD69 expression on gated MAIT cells following 16 h of incubation. The gating strategy is shown in Figure 4*A*. Consistent with its agonistic activity, 5-OP-RU induced robust CD69 upregulation, whereas none of the xanthine-based compounds elicited CD69 expression above the levels observed with the established MAIT antagonist Ac-6-FP (Fig. 4*B*).

Next, we evaluated the ability of xanthine-based compounds to inhibit the activation of primary MAIT cells induced by 5-OP-RU or the less potent MAIT agonist RL-7-Me in an overnight assay. None of the xanthine-based compounds was able to inhibit the activation of MAIT cells induced by either 5-OP-RU or RL-7-Me (Fig. 4, *C* and *D*). This lack of inhibition is consistent with the limited MR1 downregulation observed in the primary B lymphocytes when co-cultured with these ligands (Fig. 2*E*). This supports a model in which these xanthine-based ligands primarily contribute to intracellular MR1 retention rather than functional modulation of MAIT cell activity, potentially due to their lower potency in comparison to the MAIT-inhibitory riboflavin catabolites (28).

### Crystal structures of MR1-nucleotide-related ligand complexes

**Figure 5:**
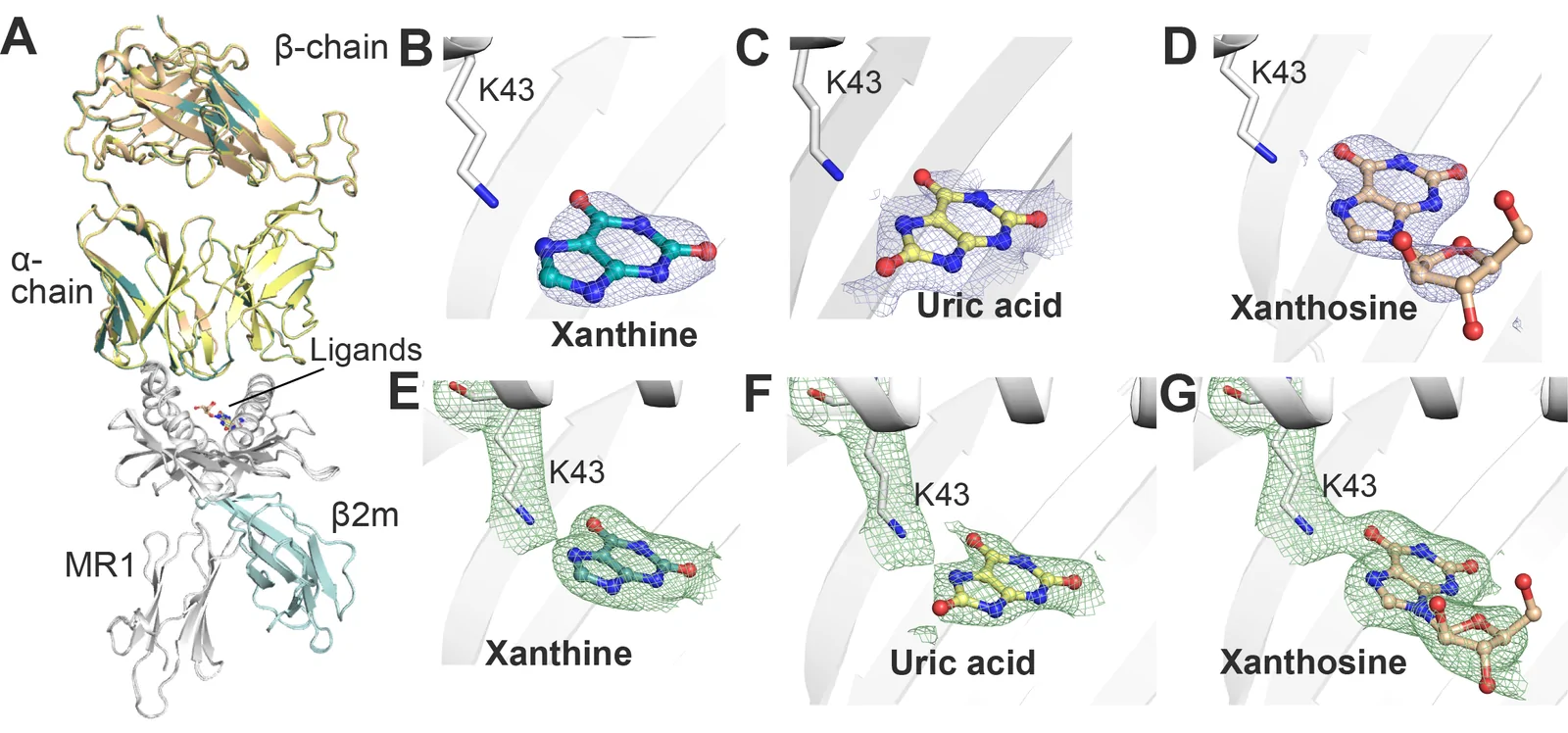
**Crystal structures of the MR1 complexes with xanthine-based compounds.***A*, superposition of the TCR-MR1-ligand crystal structures showing the nucleotide-related compounds within the MR1-binding cleft interacting with MR1-Lys43. *B–D*, the crystallographic unambiguous density omit maps of xanthine (*B*), uric acid (*C*), and xanthosine (*D*) after simulated-annealing refinement (using the phenix-refine crystallographic structure-refinement program), presented as a F_observed_ − F_calculated_ map (*blue mesh*) contoured at 1.5σ that highlight the unambiguous positions of the nucleobases within MR1 cleft. *E–G*, the crystallographic working density maps of xanthine (*E*), uric acid (*F*), and xanthosine (*G*) after simulated-annealing refinement, presented as a 2F_observed_ − F_calculated_ map (*green mesh*) contoured at 1σ. MR1 and β_2_-microglobulin (β2m) were coloured *white* and *pale cyan* respectively. TCR chains, and the ligands were coloured as follows: xanthine, *teal*; uric acid, *pale-yellow*; and xanthosine, *wheat*.

As not all MR1-binding ligands can sponsor refolding of MR1 to a sufficient amount in solution, we have established a ligand displacement protocol for loading MR1 with small molecule metabolites (23). We applied this protocol to the potential candidates (a total of 10 metabolites that did not refold MR1 in solution above the background level) using MAIT AF-7 TCRs as a crystallization aid. Three ternary TCR-MR1-ligand complexes formed crystals, which diffracted at 2 to 2.5 Å resolution. Analysis of the unbiased electron density showed that three nucleotide-related compounds, specifically xanthine, uric acid, and xanthosine, were effectively loaded into the MR1 A′-pocket (Table 1 and Fig. 5). These three TCR-MR1-ligand structures exhibited unambiguous omit and working electron densities within the MR1 A′-pocket, allowing detailed structural analysis (Fig. 5, *B*–*G*).

**Table 1:** Data Collection and Refinement Statistics

Parameters	MAIT AF-7 TCR-MR1-xanthine	MAIT AF-7 TCR-MR1-uric acid	MAIT AF-7 TCR-MR1-xanthosine
Wavelength (Å)	0.9537	0.9537	0.9322
Resolution range (Å)	46.38-2.60 (2.63-2.60)	48.28-2.85 (2.89-2.85)	48.26-2.49 (2.52-2.49)
Space group	C 1 2 1	C 1 2 1	C 1 2 1
Unit cell [a, b, c (Å) α, β, γ (°)]	216.64, 70.17, 143.71, 90, 104.47, 90	217.64, 70.16, 143.97, 90, 104.67, 90	217.30, 71.00, 143.94 90, 104.79, 90
Total reflections	206153 (6368)	217081 (6191)	257150 (7635)
Unique reflections	122238 (3902)	94365 (2895)	140853 (4172)
Multiplicity	1.70 (1.60)	2.30 (2.10)	1.80 (1.80)
Completeness (%)	96.88 (84.22)	98.52 (83.42)	96.77 (77.90)
Mean I/sigma (I)	6.15 (0.87)	4.90 (1.14)	6.42 (0.88)
Wilson B-factor	51.17	56.24	49.59
R-merge	0.1002 (0.804)	0.1804 (0.9166)	0.09168 (0.9306)
R-pim	0.09836 (0.7768)	0.1455 (0.74)	0.08599 (0.8544)
CC1/2	0.99 (0.586)	0.978 (0.422)	0.992 (0.293)
CC∗	0.998 (0.859)	0.995 (0.77)	0.998 (0.673)
R-work	0.1769 (0.3395)	0.1799 (0.2906)	0.1840 (0.3616)
R-free	0.2215 (0.4070)	0.2163 (0.3483)	0.2150 (0.3494)
Number of non-hydrogen atoms	13396	13093	13637
Macromolecules	12848	12812	12926
Ligands	73	72	94
Solvent	475	209	617
Protein residues	1605	1601	1608
RMS (bonds)	0.008	0.014	0.002
RMS (angles)	0.92	1.36	0.57
Ramachandran favored (%)	98.10	97.65	97.48
Ramachandran allowed (%)	1.77	2.29	2.46
Ramachandran outliers (%)	0.13	0.06	0.06
Average B-factor	54.60	55.24	56.75
Macromolecules	54.606	55.41	56.81
Ligands	60.86	55.33	57.80
Solvent	52.21	44.99	55.47

Statistics for the highest-resolution shell are shown in parentheses.

### The molecular basis of xanthine-based compounds binding to MR1

**Figure 6:**
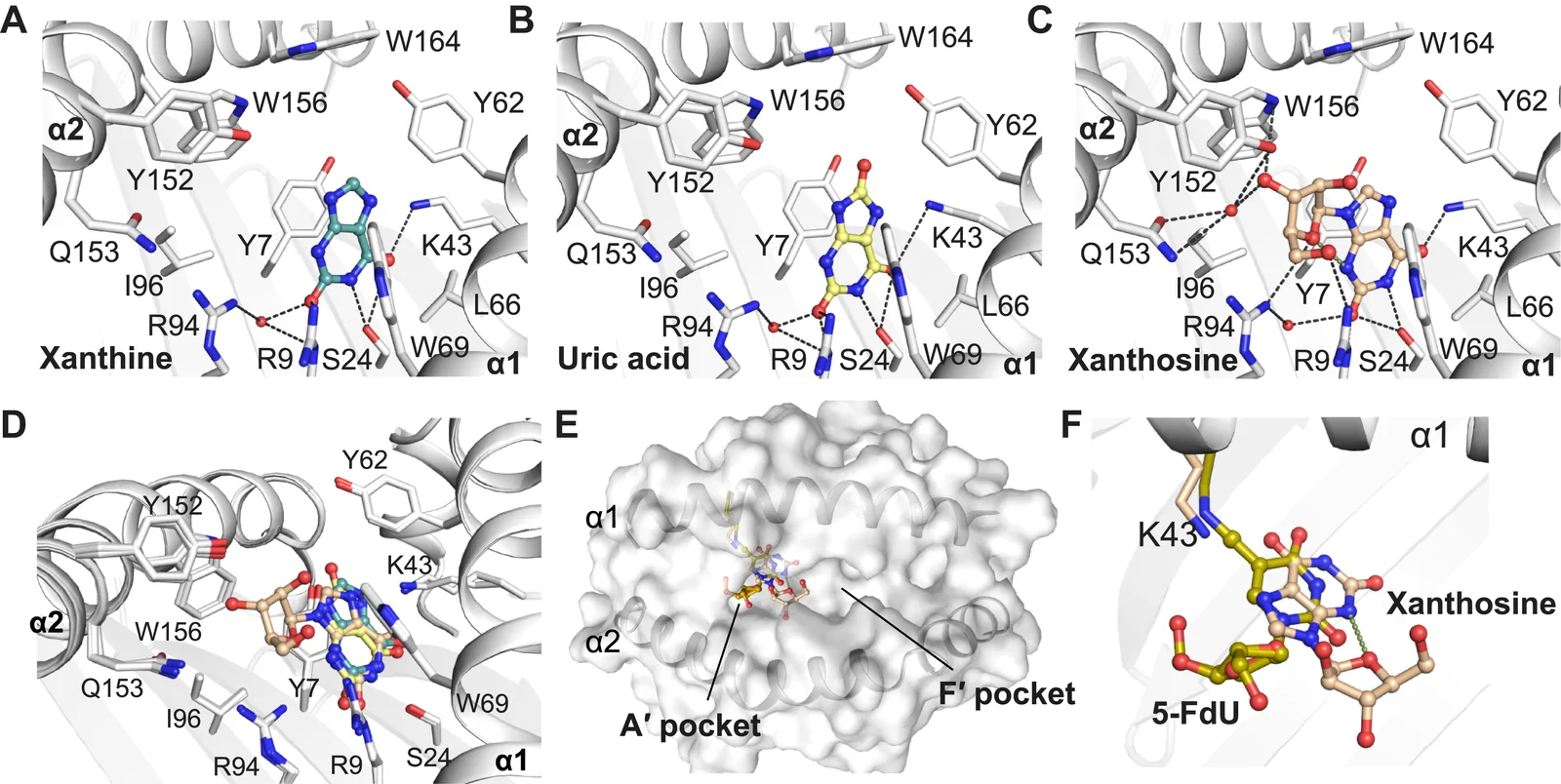
**Interactions of xanthine-based compounds within the MR1 pocket.***A–C*, The interactions between the xanthine (*A*), uric acid (*B*), and xanthosine (*C*) and the residues of MR1 A′-pocket. (*D*) Superposition of the nucleobases within the MR1-binding cleft displaying all MR1-Ags interacting residues as *white sticks*. *E*, the unique orientation of the sugar ring of the xanthosine is close to the entrance of the shallow F′-pocket of MR1. *F*, the intramolecular H-bond within the xanthosine metabolite minimises the freedom of the ribose sugars compared to the deoxyribose ring in the MR1-5-FdU structure (PDB; 9EK7). The colours are consistent with those in Figure 5 and the 5-FdU is coloured *olive-yellow*.

The three xanthine-based compounds were located within the A′-pocket of the MR1 antigen-binding cleft but did not form covalent interactions with MR1 (Fig. 6, *A*–*C*). Nevertheless, the carbonyl groups of these purine rings formed direct H-bonds with MR1-Lys43 (Fig. 6, *A*–*C*). Despite the chemical differences between these nucleobases, minimal conformational changes within the A′-pocket residues were observed, and the side chains of most MR1 ligand-binding residues were largely conserved (Fig. 6*D*). Notably, the xanthine rings in the three structures adopted similar orientations and were stabilized within the MR1 A′-pocket by hydrophobic interactions with MR1-Tyr7, Tyr62, Leu66, Ile96 and Trp69, along with a network of direct H-bonds formed between the carbonyl groups of the ring and MR1-Arg9 and Ser24, as well as water-mediated H-bonds with Arg94 (Fig. 6, *A*–*C*). The extra carbonyl group of uric acid was not involved in any contacts within the MR1 A′-pocket (Fig. 6*B*).

In the MR1-xanthosine structure, the ribose sugar was stabilized by hydrophobic interactions with MR1-Tyr62, Arg94, Ile96, Tyr152, and Trp156 (Fig. 6*C*). The three hydroxyl groups of the ribose sugar showed higher mobility compared to the backbone of xanthosine revealing their flexibility in the cleft. Yet, these hydroxyl groups formed a range of direct and/or water-mediated H-bonds with MR1-Arg9, Arg94, Gln153, Trp156 and Tyr152 (Fig. 6*C*). Consistent with its chemical structure, xanthosine does not form a covalent bond with Lys43. Although Lys43 is positioned in close proximity to the ligand, refinement of the crystallographic data supports a non-covalent interaction rather than a covalent linkage. In addition, an intramolecular H-bond was formed between the xanthosine purine ring and the carbonyl of the ribose sugar, akin to the observed intramolecular H-bond within the 5-OP-RU ligand. This bond restricts the movement of the sugar ring inside the cleft, constraining its orientation toward the entrance of the F′-pocket of MR1 (Fig. 6*E*). This raises the possibility that any modification/addition to this ribose sugar, *e*.*g*., ring phosphorylation, may orient toward the F′-pocket of MR1(Fig. 6, *E* and *F*). When compared to the structure of MR1-5-FdU, the deoxyribose of the 5-FdU was oriented in an opposed direction toward the outer side of the A′-pocket (Fig. 6, *E* and *F*). Notably, the nucleobase rings adopted dissimilar orientations in the pocket for pyrimidines and purines (Fig. 6*F*), revealing adequate malleability within the MR1 antigen-binding pocket to house distinct chemical scaffolds.

## Discussion

Host carbonyl-nucleobase adducts have recently been identified as a class of self-ligands for MR1, following the observation of enrichment in certain nucleic acid metabolic pathways under stress-related conditions (29). These endogenous adducts, *e*.*g*., M_3_Ade, mainly arise from the condensation of nucleobases and nucleosides with reactive carbonyl species, including malondialdehyde (MDA), 4-hydroxy-2-nonenal, and 4-oxo-2-nonenal (4-ONE), which are commonly generated during oxidative stress in both normal and diseased states, such as cancer or chronic degenerative diseases (42). Other MR1 binding adducts, *e*.*g*., 5-FdU, result from the direct oxidation of typical nucleobases by the reactive oxygen species (ROS) in the mitochondria (31).

Through a systematic investigation of a broad range of endogenous canonical and epigenetic purine and pyrimidine-based nucleobases, we have described compounds derived from purine metabolism that can bind MR1. Specifically, we found that MR1 can anchor purine-based compounds, such as xanthine, uric acid, and xanthosine nucleobases, within the MR1 A′-pocket, adopting distinct docking modes and interaction networks. Among the tested nucleobases, xanthosine exhibited a restrained orientation of the ribose moiety toward the entrance of the MR1 F′-pocket, suggesting that further ribose modifications may be accommodated by this unoccupied cleft.

These xanthine-derived compounds prompted a reduction in the MR1 level on the surface of MR1-overexpressing antigen-presenting cells, similar to the recently documented riboflavin catabolites (28). Despite their distinct chemical origins, both classes of host-derived metabolites bind within the MR1 antigen-binding cleft without forming the Schiff base with MR1-Lys43 and consequently reduce MR1 surface expression. Given that Schiff base formation is a key determinant of MR1 stabilization and intracellular trafficking, the inability of these ligands to covalently engage Lys43 likely results in suboptimal stabilization of MR1 and promotes its retention within the ER or targeting for degradation. This suggests that these metabolites may function as endogenous retention ligands, limiting MR1 surface availability. MR1 downregulation was seen at physiological concentrations of uric acid, which are normally close to 300 to 400 μM in human blood (43). In the case of xanthine and xanthosine, the concentrations used were higher than those found in human blood. Under normal physiologic conditions, xanthine levels in humans are less than 1 μM (44, 45); however, under hypoxic/inflammatory conditions, these levels have been reported as high as 50 to 100 μM while pH concomitantly drops below 7.0 (46). Also, it is plausible that low concentrations of purine and riboflavin catabolites, in addition to other yet uncharacterized ligands, cooperate to fine-tune MR1 surface expression, which will ultimately act as an intrinsic mechanism for MR1-dependent immune surveillance and prevent inappropriate MAIT cell activation in the absence of infection.

In contrast to the riboflavin catabolites, the reduction in cell-surface MR1 expression induced by xanthine-derived compounds did not translate into measurable inhibition of MAIT cell activation. One possible reason could be their lower affinities to MR1, as indicated by the FP binding assay, and their comparatively weaker interactions with MR1. This reduced binding affinity most likely limits the capacity of xanthine-derived compounds to effectively compete with MAIT agonists, including 5-OP-RU/RL-7-Me, for full occupancy of the MR1 antigen-binding cleft. Consequently, although xanthine-derived compounds influence MR1 intracellular trafficking and surface availability, the magnitude of this effect may be insufficient to alter the presentation of stimulatory ligands and suppress downstream MAIT cell responses. These data suggest that affinity alone is unlikely to fully account for the observed phenotype. Despite occupying the MR1 binding cleft, these ligands promote intracellular retention of MR1 and fail to activate MAIT cells, suggesting that ligand-specific structural features also influence MR1 trafficking. We therefore propose that both the strength and the nature of the MR1-ligand interaction determine whether ligand-bound MR1 is retained intracellularly or exported to the cell surface. We previously reported that despite the limited MR1 surface upregulation induced by 5-OP-RU, this ligand remains the most potent known MAIT activation signal, implying that MR1 surface level is not the only modulator of MAIT cell activity (15). An alternative explanation could be that the extent of MR1 downregulation induced by xanthine-derived compounds is less pronounced than that observed with riboflavin catabolites, resulting in a residual pool of MR1 molecules that remains available for loading and presentation of activating ligands. Together, these findings suggest that modulation of MR1 surface expression alone may not be sufficient to inhibit MAIT cell activation and that the functional outcome is likely determined by a combination of ligand affinity and the degree of MR1 trafficking blockade. Also, this does not exclude the possibility of blocking lower-affinity ligands for the non-MAIT MR1T cells.

In conclusion, we have described a group of endogenous xanthine-based compounds that bind the MR1 antigen-binding pocket and limit MR1 trafficking, thereby reducing MR1 cell surface expression. These findings expand the chemical and functional landscape of MR1 ligands and establish purine metabolism as a novel intrinsic regulator of MR1 biology. Our results suggest that MR1 continuously samples a diverse pool of host-derived metabolites, with distinct ligand classes differentially promoting MR1 stabilization or retention. This dynamic ligand-dependent regulation may represent a fundamental mechanism controlling MR1 availability, thereby preserving immune homeostasis while maintaining readiness to respond to infection, metabolic perturbation, or cellular stress.

## Experimental procedures

### Compounds tested in cellular assays and crystallography experiments

Ac-6-FP (Schircks Laboratories) was dissolved at 10 mM in water supplemented with 10 mM NaOH. 5-OP-RU was synthesized as reported previously in DMSO (47). All other compounds were obtained commercially (Sigma-Aldrich, Carbosynth, Jena Biosciences or Cayman Chemicals) and dissolved in 10 mM NaOH or DMSO.

### Fluorescence polarization (FP) assay

Various concentrations of the investigated nucleotides were incubated in competition with 10 nM TAMRA-conjugated JYM20 for binding 100 nM empty hMR1 protein in the FP assay buffer (25 mM HEPES, pH 7.5, 150 mM NaCl, 5 mM EDTA) (25). Compounds were dissolved in 10 mM NaOH or DMSO, and then diluted in FP assay buffer. The fluorescence polarization of TAMRA was measured after 24 h of incubation at 37 °C using a PHERAstar microplate reader (BMG LABTECH). The ligand-binding curves were simulated by non-linear regression with Prism software (GraphPad Software Inc.) using a sigmoidal dose-response curve. The IC_50_ binding values were used as a measurement of the binding affinity and calculated as the ligand concentration required for 50% inhibition of JYM20 binding to MR1 molecules.

### Quantification of cell surface MR1

Surface expression of MR1 following ligand exposure was assessed as previously described (17, 23). This study utilized C1R antigen-presenting B lymphocytes overexpressing MR1∗*01* (C1R.MR1), the MR1^K43A^ mutant (C1R.MR1^K43A^) (41), the MR1^R9H^ mutant (C1R.MR1^R9H^) (40), or HLA-A∗*02*:*01* (C1R-A2), along with monocytic THP-1 cells overexpressing MR1 (28). Cells (2 × 10^5^) were incubated with candidates for either 3 or 16 h at 37 °C in 5% CO_2_ in 200 μl of folate-free RPMI 1640 medium (Gibco, Cat. No. 11875-093), supplemented with 10% fetal bovine serum, sodium pyruvate (1 mmol/L), HEPES (15 mmol/L; pH 7.2–7.5), penicillin (100 U/ml), streptomycin (100 μg/ml), GlutaMAX (2 mmol/L), non-essential amino acids (0.1 mmol/L) (all from Thermo Fisher Scientific, Life Technologies), and 2-mercaptoethanol (50 μmol/L; Sigma-Aldrich) (RF-10). Following incubation, cells were stained with near-infrared (NIR) live/dead stain (LIVE/DEAD Fixable Near-IR Dead Cell Stain Kit; Thermo Fisher Scientific, Life Technologies) and subsequently incubated with biotinylated 8F2.F9 anti-MR1 antibody for 30 min on ice. Excess antibody was removed by washing with PBS containing 2% fetal bovine serum (hereafter referred to as 2% FACS buffer). Cells were then labelled with PE-conjugated streptavidin (30 μg/ml; BioLegend), followed by additional washes with 2% FACS buffer. Surface MR1 expression was quantified based on PE fluorescence intensity. Data acquisition was performed using an LSRFortessa X-20 flow cytometer (BD Biosciences) with Diva software (BD Biosciences), and subsequent analysis was conducted using FlowJo software (BD Biosciences).

### PBMC preparation and staining

Healthy donor blood packs were obtained through the Australian Red Cross LifeBlood Service after approval from the Melbourne University Human Research Ethics Committee (ethics number: 30058) and peripheral blood mononuclear cells (PBMCs) were prepared by separation on Ficoll-Paque Premium (GE Healthcare). Cells were frozen in FCS containing 10% DMSO and stored in liquid nitrogen until use. Upon thawing, cells were washed, then rested for 2 h before treatment. 2 × 10^5^ PBMCs were titrated with xanthine-based compounds for 16 h with and without 5-OP-RU or RL-7-Me. After treatment, cells were then stained with antibodies to CD3 (BioLegend; 1:100), CD69 (BioLegend; 1:100) and TRAV1-2 (BioLegend; 1:100) as well as human MR1-5-OP-RU tetramers (BV421, prepared in house) and NIR live/dead stain (LIVE/DEAD Fixable Near-IR Dead Cell Stain Kit; Thermo Fisher Scientific, Life Technologies) for 30 min and were then fixed with 1% paraformaldehyde before analysis on a BD LSR Fortessa Flow Cytometer (BD Biosciences). Data were analyzed using FlowJo software (Treestar, v10).

### Expression and preparation of denatured inclusion bodies of MR1 and TCRs proteins

Human TRAV1-2/TRAJ33, TRBV6-1, MR1, and β2m genes were expressed for 3 to 4 h in BL21 *E. coli* after induction with 1 mM isopropyl b-D-1-thiogalactopyranoside as described previously (4). *E. coli* cells were pelleted and resuspended in a buffer containing 50 mM Tris-HCL, 10 mM DTT, 1 mM EDTA pH 8.0 and 25% (w/v) sucrose. Then, the inclusion bodies (IB) proteins were extracted by lysis of bacteria in a buffer containing 50 mM Tris-HCL pH 8.0, 10 mM DTT, 1% (w/v) Triton X-100, 1% (w/v) sodium deoxycholate, 100 mM NaCl, 5 mM MgCl_2_ and 1 mg DNase-I per liter of starting culture. The IB protein was homogenized with a polytron homogenizer, centrifuged and washed sequentially with (1) a buffer containing 50 mM Tris pH 8.0, 1 mM DTT, 0.5% Triton X-100, 100 mM NaCl and 1 mM EDTA, and (2) a buffer containing 50 mM Tris pH 8.0, 1 mM DTT and 1 mM EDTA. The IB was then solubilized in a buffer containing 2 mM DTT, 20 mM Tris pH 8.0, 6 to 8 M urea and 0.5 mM EDTA. This suspension was then centrifuged, and the supernatant containing solubilized, denatured IB protein was stored at 80 °C.

### Refolding and purification of MR1 and MAIT TCR

Human A-F7 (TRAV1-2/TRBV6-1) MAIT TCR protein was refolded from the separate α- and β-chain inclusion bodies at 4 °C overnight in the refolding buffer containing 0.1 M Tris pH 8.5, 6 M urea, 2 mM EDTA, 0.4 M L-arginine, 0.5 mM oxidized glutathione and 5 mM reduced glutathione as described previously (20). The wild-type MR1-β2m was refolded in the same refolding buffer, along with the 10× molar ratio of the investigated compound as described previously (17). Refolded MR1-ligand and TCR proteins were purified by three sequential purification steps: crude DEAE anion exchange, S200 15/60 size exclusion chromatography and HiTrap-Q HP anion exchange. The protein quality and purity were then assessed using sodium dodecyl sulphate–polyacrylamide gel electrophoresis (SDS-PAGE), concentrated and further quantified using a NanoDrop spectrophotometer.

### Crystallization, data collection, structure determination, and refinement

Purified A-F7 (TRAV1-2/TRBV6-1) TCR was mixed with MR1-β2m-Ag in a 1:1 M ratio at a concentration of 4 to 6 mg/ml and kept on ice for 2 h. The hanging-drop, vapor diffusion method at 20 °C was utilized to produce A-F7 TCR-MR1-Ag crystals with a precipitant reservoir solution consisting of 100 mM Bis–Tris propane (BTP; pH 6.0–6.7), 10 to 20% PEG 3350, and 200 mM sodium acetate, as reported previously (17). The ternary complexes crystals grew in one week, and then they were harvested, quickly soaked in reservoir solution with 10 to 14% glycerol for cryoprotection and flash-frozen in liquid nitrogen. X-ray diffraction data sets were collected at 100 K on the Australian Synchrotron at either MX1 or MX2 beamlines. Diffraction data were processed using XDS (48) and programs from the CCP4 suite (49) and Phenix package (50). The previously solved A-F7 TCR and MR1 structure (PDB, 4L4T (19)) was used as a search model for molecular replacement in PHASER program to solve the ternary structure of TCR-MR1-purine catabolite complexes. Model building in COOT (51), followed by iterative rounds of refinement using Phenix.refine (50), was conducted, and the models were validated using MolProbity (52). Geometry restraints for the ligand were generated using the Grade web server (Global Phasing Ltd), and the resulting restraint dictionary (.cif) was used during crystallographic refinement (53). The final refinement statistics of the TCR-MR1-purine catabolite structures are summarized in Table 1. The contacts were generated by the Contact program from the CCP4 suite (49). Molecular graphics representations were generated using PyMOL Molecular Graphics System, Version 2.2, (Schrödinger, LLC, New York, NY).

## Data and code availability

The atomic coordinates of A-F7 TCR-MR1 in complex with xanthine, uric acid and xanthosine, along with associated structure factors, have been deposited at the protein data bank (www.rcsb.org) with accession codes 26SJ, 26SK, and 26SL respectively.

## Supporting information

This article contains supporting information.

## Conflict of interest

J. R., J. M., D. P. F., J. Y. W. M. and A. J. C. are named inventors on patent applications (PCT/AU2013/000742, WO2014005194) (PCT/AU2015/050148, WO2015149130) describing MR1 ligands and MR1 tetramers. The other authors declare no competing interests.
