# Supplementary material for: The MHC-I related protein 1 MR1 can bind host purine catabolites
Source: J Biol Chem. 2026 Aug 12;302(10):113428. doi: 10.1016/j.jbc.2026.113428 (PMC13597202; doi:10.1016/j.jbc.2026.113428)
Supplement: Supplementary file 1 [file mmc1.docx]

**The MHC-I related protein 1 MR1 can bind host purine catabolites**

Mohamed R. Abdelaal^1^, Xin Yi Lim^2^, Jeffrey Y.W. Mak^3^, David P. Fairlie^3^, James McCluskey^2^, Alexandra J. Corbett^2^, Nicholas A. Gherardin^2^, Wael Awad^1#^ & Jamie Rossjohn^1#^

^1^Department of Biochemistry and Molecular Biology, Immunity Program, Biomedicine Discovery Institute, Monash University, Clayton, Australia

^2^Department of Microbiology and Immunology, The University of Melbourne at the Peter Doherty Institute for Infection and Immunity, Melbourne, Australia

^3^Centre for Chemistry and Drug Discovery and ARC Centre of Excellence for Innovations in Peptide and Protein Science, Institute for Molecular Bioscience, The University of Queensland, Brisbane, Queensland, Australia

\# joint senior authors

Correspondence: jamie.rossjohn@monash.edu

**Running title:** MR1 presents nucleobase ligands

**Keywords:** MR1, mucosal-associated invariant T cell, antigen presentation, nucleobases.

**Supplementary Figures**

**Figure S1. Functional response to nucleotide-related candidates in cell co-culture assays. (A-C)** C1R cells were co-cultured with Jurkat.MAIT cells (1 x 10^5^ each) and titrating concentrations of compounds (L🡪R: 200, 40, 8, 1.6 μM) for 3 h. Appropriate solvent controls are included (DMSO for all compounds except; dUMP, dTMP (water) and xanthine, uric acid (NaOH). Cells were then stained with antibodies to MR1, CD3, CD69 and MHC Class I and 7AAD, before analysis by flow cytometry, to detect the surface expression of **(A)** MR1 and **(B)** MHC Class I expression on C1R cells, as well as **(C)** CD69 expression on the surface of Jurkat.MAIT cells. Data in **A-C** are from two independent experiments performed in duplicates.

**Figure S2. Cellular screening of xanthine-based compounds. (A)** Depicted is the proportion of viable cells after exposure to purine catabolites overnight, indicating no loss in cellular viability. **(B)** C1R cells overexpressing HLA*02:01 were incubated for 16 h with titrated quantities of purine catabolites, followed by flow cytometry. **(C)** Gating strategy for MR1 measurement on the surface of PBMC-derived B-lymphocytes. Data in **B** represent the fold change of MFI from three independent experiments performed in duplicates, with standard error (SEM) represented by error bars. One-way ANOVA statistical analysis was performed for all samples, with Dunnett’s multiple comparisons performed using solvent as a control for the comparison (ns: not significant; P > 0.05). MFI, geometric mean fluorescence intensity.
